# Differences between the global transcriptomes of *Salmonella enterica* serovars Dublin and Cerro infecting bovine epithelial cells

**DOI:** 10.1186/s12864-022-08725-z

**Published:** 2022-07-08

**Authors:** Serajus Salaheen, Seon Woo Kim, Bradd J. Haley, Jo Ann S. Van Kessel

**Affiliations:** Environmental Microbial and Food Safety Laboratory, Beltsville Agricultural Research Center, USDA-ARS, Beltsville, MD USA

**Keywords:** RNA-Seq, *Salmonella*, Serovar, *S.* Dublin, *S.* Cerro, Epithelial cells

## Abstract

**Background:**

The impact of *S. enterica* colonization in cattle is highly variable and often serovar-dependent. The aim of this study was to compare the global transcriptomes of highly pathogenic bovine-adapted *S. enterica* serovar Dublin and the less pathogenic, bovine-adapted, serovar Cerro during interactions with bovine epithelial cells, to identify genes that impact serovar-related outcomes of *S. enterica* infections in dairy animals.

**Result:**

Bovine epithelial cells were infected with *S. enterica* strains from serovars Dublin and Cerro, and the bacterial RNA was extracted and sequenced. The total number of paired-end reads uniquely mapped to non-rRNA and non-tRNA genes in the reference genomes ranged between 12.1 M (Million) and 23.4 M (median: 15.7 M). In total, 360 differentially expressed genes (DEGs) were identified with at least two-fold differences in the transcript abundances between *S*. Dublin and *S*. Cerro (false discovery rate ≤ 5%). The highest number of DEGs (17.5%, 63 of 360 genes) between the two serovars were located on the genomic regions potentially associated with *Salmonella* Pathogenicity Islands (SPIs). DEGs potentially located in the SPI-regions that were upregulated (≥ 2-fold) in the *S*. Dublin compared with *S*. Cerro included: 37 SPI-1 genes encoding mostly Type 3 Secretion System (T3SS) apparatus and effectors; all of the six SPI-4 genes encoding type I secretion apparatus (*siiABCDEF*); T3SS effectors and chaperone (*sopB*, *pipB*, and *sigE*) located in SPI-5; type VI secretion system associated protein coding genes (*sciJKNOR*) located in SPI-6; and T3SS effector *sopF* in SPI-11. Additional major functional categories of DEGs included transcription regulators (*n* = 25), amino acid transport and metabolism (*n* = 20), carbohydrate transport and metabolism (*n* = 20), energy production and metabolism (*n* = 19), cell membrane biogenesis (*n* = 18), and coenzyme transport and metabolism (*n* = 15). DEGs were further mapped to the metabolic pathways listed in the KEGG database; most genes of the fatty acid *β*-oxidation pathway were upregulated/uniquely present in the *S*. Dublin strains compared with the *S*. Cerro strains.

**Conclusions:**

This study identified *S. enterica* genes that may be responsible for symptomatic or asymptomatic infection and colonization of two bovine-adapted serovars in cattle.

**Supplementary Information:**

The online version contains supplementary material available at 10.1186/s12864-022-08725-z.

## Background

*Salmonella enterica* is a major cause of infection-related morbidity among humans and domesticated animals worldwide. Although, a small percentage of the > 2600 known serovars account for most human infections, all *S. enterica* serovars are considered potential human pathogens [1, 2]. Many serovars can asymptomatically infect food-producing animals and these animals can shed the bacterium in their feces, thereby potentially contaminating foods, other animals, humans, and the environment causing an under-appreciated public health risk [3, 4].

Dairy cows are known reservoirs of *S. enterica* and the impact and/or severity of infection in cattle is highly variable and often serovar-dependent [2, 5–9]. For example, serovars Dublin and Typhimurium are generally associated with salmonellosis in dairy calves and adult cows, causing mild illnesses to severe systemic infections. Serovars Cerro, Kentucky, Montevideo, Mbandaka, and Senftenberg, among others, have been recovered from sick animals, but they have also been frequently reported to be harbored and shed by apparently asymptomatic animals [4, 6, 10, 11]. Among the *Salmonella* serotyping submissions received at the National Veterinary Services Laboratories, Ames, IA, in 2016, a total of 332 of the 1603 clinical isolates from cattle belonged to serovar Dublin followed by Cerro (275 isolates) and Typhimurium (142 isolates), while a higher number of non-clinical isolates (from the herd and flock monitoring programs, environmental sources, food, and others) belonged to serovar Cerro (30 of 290 isolates) followed by Typhimurium (25 isolates) and Montevideo (17 isolates) [11]. At the beginning of a longitudinal study (six years) of an endemically infected dairy herd, we occasionally identified a pathogenic serovar, Typhimurium, and then described a long-term outbreak (over three years) of serovar, Cerro. The Cerro outbreak was gradually supplanted by Kentucky and this serovar established in the herd for over a year. These serovars were persistent in greater than 50% of the herd for extended periods of time without observable clinical symptoms or production loss [4, 6]. Further work determined that Cerro and Kentucky were widespread in the dairy farms surrounding this study farm and apparently endemic in the region [12].

To understand why some serovars cause severe and sometimes fatal infections, while others apparently asymptomatically colonize cattle as commensal members of the gut community, we investigated the interactions (association and invasion) of *S. enterica* strains from 13 different serovars with bovine mammary epithelial cells [13]. We observed that *S*. *enterica* serovar Dublin (*S*. Dublin) strains were the most invasive, while *S*. Kentucky, *S*. Mbandaka, *S*. Cerro, and *S*. Give strains were the least invasive under cell-culture conditions. Recent research has also identified *S*. Dublin as the most invasive serovar compared with serovars Heidelberg, Mbandaka, and Typhimurium when infecting a bovine ileal epithelial cell line [14].

Currently, there are significant knowledge gaps in our understanding of genomic and transcriptomic features that are responsible for the differential pathogenicity and colonization of *S. enterica* serovars in the bovine host. *S. enterica* harbors an array of genes that aid in its attachment, invasion, and persistence in specific hosts [15, 16]. For example, the type III secretion systems (T3SS) that are encoded in *Salmonella* pathogenicity islands: SPI-1 and SPI-2, and a type I secretion apparatus encoded in SPI-4 are apparently necessary for *S*. Typhimurium colonization in cattle but have little impact on the colonization of chicks [16] highlighting the importance of studying *S. enterica* infectibility on species-specific infection models. The aim of this study was to compare the global transcriptomes of the highly pathogenic bovine-adapted *S*. Dublin and the less pathogenic bovine-adapted *S*. Cerro during interactions with bovine epithelial cells to identify genes impacting serovar-related outcomes of *S. enterica* infections in cattle.

## Methods

### Bacterial strains, bovine epithelial cells, and growth medium

Four representative wild type *S. enterica* strains that were previously isolated from dairy (bulk tank milk or bulk tank milk filters) were included for RNA-Seq analysis [17]. Two strains were serovar Dublin and two strains were serovar Cerro. Both *S*. Cerro strains in this study were susceptible to all of the antimicrobials on the NARMS GN panel (Sensititre™ NARMS Gram Negative Plate, Catalog number: CMV3AGNF, Thermo Scientific™, Waltham, MA) and both *S*. Dublin strains were resistant to more than three classes of antimicrobials [18]. Using an association-invasion assay we previously observed that these *S*. Dublin and *S*. Cerro strains had different association and invasiveness towards bovine epithelial cells [13].

For *in vitro* RNA isolation, frozen stocks (-80 °C in 20% (v/v) glycerol) of bacterial cultures were grown on Luria–Bertani (LB, Acumedia, Neogen Culture Media, Lansing, MI; contained approximately 5 g NaCl per liter) agar overnight at 37 °C. For RNA extraction from *S. enterica* strains associated with bovine epithelial cells, a single colony from the overnight culture was inoculated into LB broth and grown at 37 °C for 16 h. The continuous immortalized culture of bovine mammary epithelial cell-line, MAC-T, was maintained in 75-cm^2^ cell culture flasks in Dulbecco’s modified Eagle medium (DMEM; Cellgro, Manassas, VA) containing 10% heat-inactivated fetal bovine serum (FBS, Sigma-Aldrich, St. Louis, MO) and 100 μg/mL of gentamicin (Sigma-Aldrich, St. Louis, MO) at 37 °C under 5% CO_2_ humidified atmosphere [19].

### RNA isolation from ***S. enterica*** associated with bovine epithelial cells

Approximately 2 × 10^6^ MAC-T cells were seeded in 75 cm^2^ cell culture flasks and infected with each *S. enterica* strain at a multiplicity of infection (MOI) of 10:1 (bacteria: host) [13]. After infection for 2 h, unattached bacterial cells were removed by washing three times with Phosphate Buffered Saline (PBS, 1 × , pH 7.4). The infected MAC-T cells were lysed in ice-cold RNA stabilization solution [0.2% SDS (prepared fresh on the day of the experiment), 19% ethanol, 1% acidic phenol in water] for 30 min [20] to halt transcription and stabilize the bacterial mRNA [21]. The lysates containing associated bacterial cells that remained attached to and invaded into MAC-T cells were harvested using centrifugation at 25,000 × g at 4 °C for 1 min. Bacterial pellets were washed three times with ice-cold PBS. Bacterial pellets originating from multiple cell culture flasks were pooled and then processed for total RNA extraction using a Quick-RNA Fungal/Bacterial Miniprep Kit (Zymo Research, Irvine, CA) according to the manufacturer’s protocol. Total RNA was extracted from three biological replicates for each *S. enterica* strain. We incorporated the optional bead-beating step recommended by the manufacturer during RNA extraction and observed that extending the duration of bead-beating increased total RNA yield. In-column DNase treatment was included during RNA extraction (Zymo Research, Irvine, CA). RNA samples were subjected to a second in-solution DNase treatment to remove any residual genomic DNA using Ambion™ DNase I according to the protocol provided by RiboPure™ RNA Purification Kit (Ambion, Invitrogen, Carlsbad, CA). RNA concentration was measured with Qubit using an RNA HS Assay Kit (Invitrogen™, Thermo Scientific™, Waltham, MA). RNA quality was determined with Agilent Bioanalyzer 2100 using RNA Pico Chip (Agilent, Santa Clara, CA).

### Removal of host-RNA contaminants, library preparation, and RNA sequencing

Total RNA was treated with the MICROBEnrich™ Kit (Invitrogen™, Thermo Scientific™, Waltham, MA) to eliminate/reduce MAC-T cell RNA contaminants. rRNA was depleted from total RNA of each sample using Illumina Ribo-Zero Plus rRNA Depletion Kit (Illumina, San Diego, CA) according to the manufacturer’s instructions. The extent of rRNA depletion from the RNA samples was assessed using the Agilent Bioanalyzer 2100 as described above. Twelve RNA-Seq libraries (three biological replicates for each of four *S. enterica* strains belonging to serovars Dublin and Cerro) were prepared using the TruSeq® Stranded mRNA Library Prep kit (Illumina, San Diego, CA) following the manufacturer’s instructions. Paired-end sequencing (2 × 150 bp reads) was conducted with a NextSeq 500/550 v2.5 high-output flow cell on a NextSeq500 sequencing platform (Illumina, San Diego, CA).

### RNA-Seq data analyses

For data analyses, default parameters were used for all software unless otherwise specified. Sequence data were demultiplexed using the BCL2FastQ v2.15.0.4 program (Illumina). Reads were trimmed and sequencing adapters were removed using Trimmomatic v0.36 (leading 20, trailing 20, sliding 4:20, min len 36) [22]. Only paired reads were retained for further analyses. Cleaned and curated RNA-Seq reads were aligned to the bovine reference genome (*Bos taurus* UMD3.1 genome assembly) using Spliced Transcripts Alignment to a Reference (STAR) aligner v2.7.2b [23]. Reads that mapped uniquely to the bovine reference genome were removed before further analyses. Sequencing reads were then mapped to serovar-specific *S. enterica* reference genomes using Bowtie 2 v2.3.2 [24]. Reads that originated from *S*. Dublin strains were mapped to *S. enterica* serovar Dublin strain USMARC-69838 chromosome and plasmid pSDU1-USMARC-69838 (GenBank accession numbers: NZ_CP032449.1, NZ_CP032450.1, *S*. Dublin reference genome henceforth). Reads that originated from *S*. Cerro strains were mapped to *S. enterica* serovar Cerro strain CFSAN001588 chromosome and plasmids pCFSAN001588_001 and pCFSAN001588_002 (GenBank accession numbers: NZ_CP012833.1, NZ_CP012834.1, NZ_CP012835.1, *S*. Cerro reference genome henceforth). The number of reads that were uniquely mapped to coding sequences of respective bacterial reference genomes was calculated using featureCounts from the Subread package v2.0.1 [25]; multi-mapping reads were not counted. For differential expression analysis, a comparison matrix was prepared by clustering the genes from the two *S. enterica* reference genomes using get_homologues_est [26] followed by BLASTn, and manual curation. This way, 3908 core genes were identified from the two-reference genomes. Details of the method for clustering homologous genes from the two reference genomes has been described in Additional file 1: Table S1.

Differential expression of genes between strains belonging to serovars Dublin and Cerro was conducted using DESeq2 in R v4.0.3 [27]. *P*-values were adjusted using the Benjamini–Hochberg method to control the false discovery rate (P_*adj*_). Genes that had  ≥ 2-fold differences in the normalized transcript abundances between the two serovars with a P_*adj*_ value of < 0.05 were considered differentially expressed genes (DEGs) [28]. To increase confidence in this data, the DEG profiles were further validated using another well-established normalization method, edgeR in R v4.0.3 [29].

Functional categories (Clusters of Orthologous Groups, COGs) of the DEGs were determined using eggNOG-mapper v2 [30]. The virulence factor (VFs) encoding genes in the two reference genomes were identified using BLASTn v2.7.1 and the core dataset of the virulence factor database (VFDB) with an identity cut-off of 95% [31]. The core dataset of VFDB includes genes associated with experimentally verified VFs. Genes of the two reference genomes that were potentially located on *Salmonella* pathogenicity islands (SPIs) were determined using BLASTn v2.7.1 and 21 SPI reference sequences (SPI-1 to SPI-21) listed by Hsu et al. [32]. Genes that were located on potential prophage regions in the reference genomes were identified using PHASTER: PHAge Search Tool Enhanced Release (region positions of only “Intact” matches were used) [33]. To identify relevant metabolic pathways listed in the Kyoto Encyclopedia of Genes and Genomes (KEGG) database, KEGG Orthology (KO) identifiers of the DEGs/serovar-specific unique genes were determined using BlastKOALA and KofamKOALA tools available from the KEGG web server [34]. KO identifiers were then mapped to the KEGG pathway database using the KEGG Mapper tool [34] followed by manual inspection. Pathways consisting of the DEGs/serovar-specific unique genes that were identified using the above method were explored from the list of metabolic pathways constructed specifically for *S. enterica* serovar Dublin strain USMARC-69838 on the BioCyc Database Collection [35].

### Quantitative RT-PCR

Quantitative reverse transcription-PCR (qRT-PCR) was conducted to validate the gene expression profiles obtained from the RNA-Seq following a previously described methodology [36] using PerfeCTa SYBR Green FastMix (Quantabio, Beverly, MA) on a Stratagene Mx3005P (Agilent Technologies, Santa Clara, CA). qRT-PCR was performed targeting three genes, *rpoD*, *invA*, and *iroB*, using the same RNA samples that were used for RNA-Seq [37]. Sequences of the primers are available from literature [38]. The relative expression/fold-changes of *invA* and *iroB* between *S*. Dublin and *S*. Cerro strains were calculated by the comparative CT method [39] using *rpoD* as the housekeeping gene.

## Results and discussion

A previous study identified differences in the abilities of *S. enterica* serovars (including Dublin and Cerro) to associate and invade cultured bovine epithelial cells [13]. Two strains from each serovar were used to compare the association and invasiveness in the previous study. The *S*. Dublin strains associated with and invaded into the bovine epithelial cells at a significantly higher level compared with the *S*. Cerro strains [13]. When the ratios of log_10_ transformed associated or invaded *S. enterica* cells and the initial inoculum size (termed as normalized interaction) were determined, the *S*. Dublin strains had a mean normalized interaction of 0.76 for both association and invasiveness, whereas the *S*. Cerro strains had a mean normalized interactions of 0.64 and 0.59 for association and invasiveness, respectively. Unlike the *S*. Cerro strains, approximately 100% of the attached *S*. Dublin cells also invaded the bovine epithelial cells, indicating that *S*. Dublin strains tended to invade once they formed stable attachments to the bovine cells. The genomic characteristics responsible for these differences are not well known. Using these same dairy farm strains of *S.* Dublin and *S*. Cerro, a comparative transcriptomic analysis was performed to identify signatures in their global transcriptomes that may be responsible for the observed differences in association and invasion. When RNA was extracted and sequenced from *S. enterica* strains that were associated (bacterial cells that were both attached to and invaded into the host cells) with bovine epithelial cells, the total number of raw paired end reads (fragments) in each sample ranged between 29.8 M and 40.7 M (median: 35.2 M). The percentages of reads that were uniquely mapped to the host genome (*Bos Taurus*) ranged from 22.1% to 43.7% for *S*. Dublin assays and 34.3% to 50.7% for *S*. Cerro assays. After removing reads that were mapped to the bovine genome, the total number of cleaned and curated reads that were mapped to the *S*. Dublin and *S*. Cerro reference genomes ranged between 16.1 M and 25.5 M (median: 17.5 M). The total number of reads that were uniquely mapped to non-rRNA and non-tRNA genes in the *S*. Dublin and *S*. Cerro reference genomes ranged between 12.1 M and 23.4 M (median: 15.7 M). Sequence depth is an important metric for analysis of transcriptomic studies and Haas et al. [40] concluded that a sequencing depth of 5–10 million non-rRNA fragments is required to profile most of the transcriptional activity for bacteria grown under diverse culture conditions. Therefore, for this study, sequencing depth and the number of non-rRNA reads that were retained after removing host-specific reads, provided sufficient coverage of the transcriptomes to conduct the desired analysis.

For RNA-Seq data analysis, genes that had at-least one mapped read-pair in each of the three technical replicates from a strain were retained for further analyses. Transcriptomic reads were mapped to 4738 (of 4826) genes in the *S*. Dublin reference, and 4326 (of 4490) genes in the *S*. Cerro reference. For differential expression analysis between the two serovars, the individual strains within each serovar served as independent biological replicates for that serovar and read counts from replicates within each strain were merged (considered as technical replicates for this analysis). When log2-transformed TPM (Transcripts Per Kilobase Million)-normalized expressions were compared, individual strains within serovars Dublin and Cerro demonstrated Spearman’s correlation coefficient values > 0.92 and > 0.97 (*p* < 0.01), respectively, indicating high reproducibility within biological replicates [41]. In addition, Spearman’s correlation coefficient values of comparisons between log2-transformed TPM from three technical replicates for individual strains were > 0.98 (*p* < 0.01) indicating high reproducibility within technical replicates. Considering the genomic diversity among strains within an *S. enterica* serovar, it is important to note that two strains may not represent the entirety of a serovar and findings from this study may only be translated to strains with similar genomic profiles especially strains that were isolated from dairy animals. Among the 3908 core genes between the two reference genomes, 521 were identified as DEGs that had at-least 2-fold differences in the transcript abundances between *S*. Dublin and *S*. Cerro strains using DESeq2 (P_*adj*_ < 0.05), while edgeR identified 374 (of 3908) genes to be DEGs with at-least 2-fold differences in transcript abundances (P_*adj*_ < 0.05). Yendrek et al. [42] reported that there may be high variability between methods for determining differential expression of RNA-Seq data and suggested that several bioinformatics tools for identifying DEGs should be used. In this study, a conservative list of 360 DEGs was obtained by integrating the DESeq2 and edgeR outputs and incorporating only the overlapping genes. Among the DEGs, 212 were upregulated in *S.* Dublin strains (compared with *S*. Cerro strains) and 148 were upregulated in *S*. Cerro strains (compared with *S*. Dublin strains). A complete list of the DEGs with respective TPM values and fold changes are presented in supplemental document, Additional file 2: Table S2.

When qRT-PCR was used to validate the gene expression profiles, transcription of two genes (*invA* and *iroB*) were examined while using *rpoD* as the housekeeping gene. RNA-Seq analyses revealed that *rpoD* was not differentially expressed between *S*. Dublin and *S*. Cerro strains but the *invA* gene was upregulated in the *S*. Dublin strains compared with the *S*. Cerro strains by 14.4-fold and 4.83-fold using RNA-Seq and qRT-PCR, respectively. The *iroB* gene was upregulated in the *S*. Cerro strains compared with the *S*. Dublin strains by 2.17-fold and 1.6-fold using RNA-Seq and qRT-PCR, respectively. Albeit to different extents, both RNA-Seq and qRT-PCR demonstrated comparable differential expression trends for the selected genes.

### Functional categories of the DEGs

**Fig. 1:**
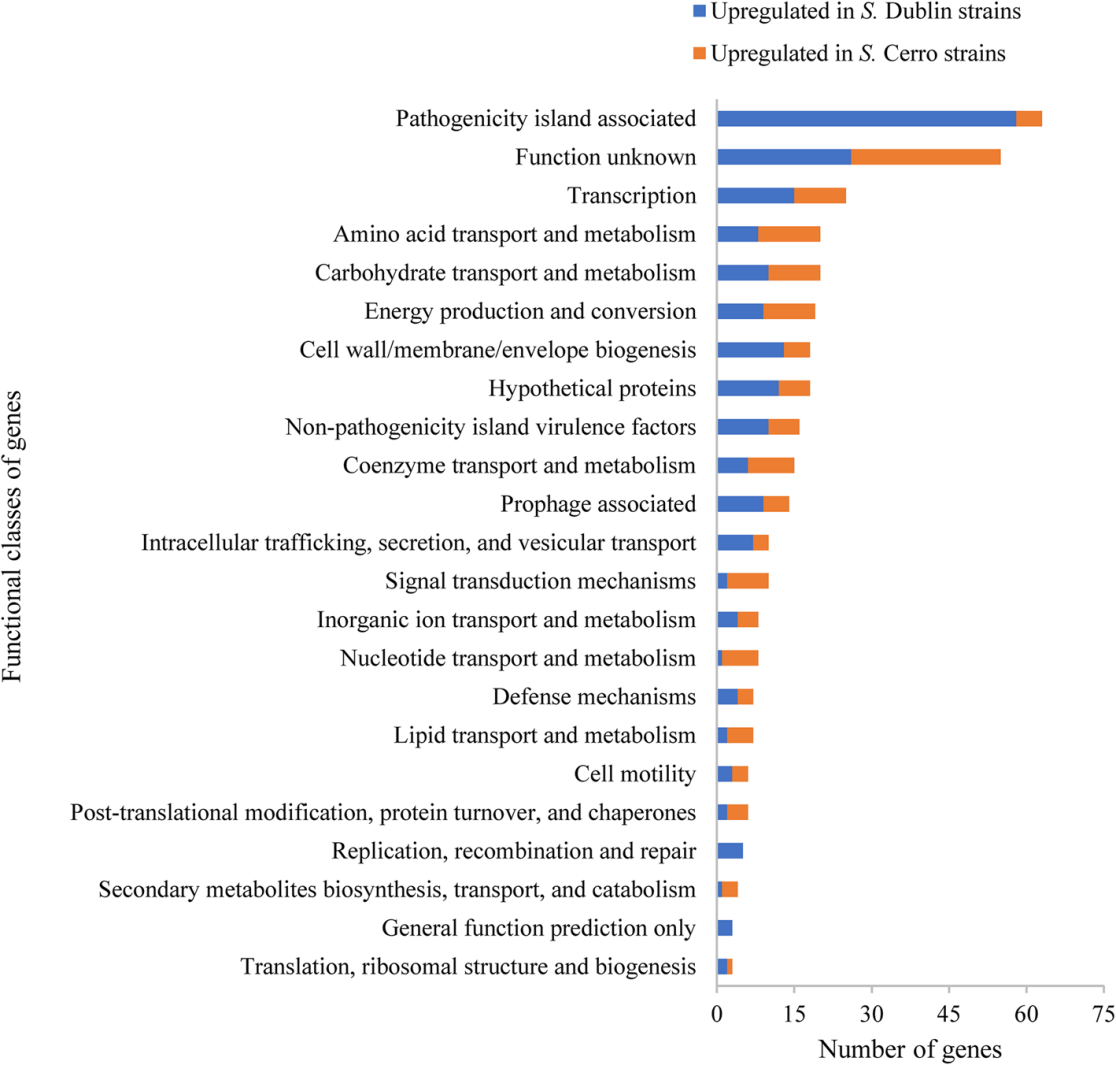
Functional classes of genes that were upregulated (by ≥ 2-fold) in the *S*. Dublin strains compared with the *S*. Cerro strains, and vice versa, during interaction with bovine epithelial cells

The genes that were differentially expressed between strains from serovars Dublin and Cerro were categorized into functional groups (Fig. 1).

In total, 63 of 360 DEGs (17.5%) between strains from the two serovars were located on the genomic regions potentially associated with SPIs. Of the 63 DEGs located on potential SPIs, 58 were more highly expressed in the *S*. Dublin strains than in the *S*. Cerro strains and only 5 of the DEGs were expressed at a higher level in the *S*. Cerro strains than in the *S*. Dublin strains. Additionally, 16 virulence factors (VFs) that were located in the *S. enterica* genomes beyond the SPI regions were differentially expressed in the *S*. Dublin and the *S*. Cerro strains. Six of those differentially expressed VFs were upregulated in the *S*. Cerro strains and were associated with biogenesis of fimbriae (the Type I fimbriae *fim* cluster). Other major functional categories of DEGs included transcription regulators (*n* = 25), amino acid transport and metabolism (*n* = 20), carbohydrate transport and metabolism (*n* = 20), energy production and metabolism (*n* = 19), cell membrane biogenesis (*n* = 18), coenzyme transport and metabolism (*n* = 15), and prophage associated genes (*n* = 14). A large number of DEGs (in total 21.1% or 76 of 360 genes) could not be categorized into specific functional groups (e.g., genes encoding uncharacterized, poorly characterized, or hypothetical proteins). The SignalP 5.0 server predicts the presence of signal peptides in bacterial, archaeal, and eukaryotic proteins (e.g., standard and lipoprotein secretory signal peptides transported by the Sec translocon and Tat signal peptides transported by the Tat translocon) with the location of their cleavage sites [43]. When the translated proteins of the uncategorized DEGs in the *S*. Dublin and *S*. Cerro transcriptomes were investigated for possible signal peptides using SignalP 5.0., 15 Sec-dependent proteins were predicted among the translated proteins of the 76 uncategorized DEGs (Additional file 3: Table S3).

In addition, approximately 3–5% of the mapped paired-end reads in each sample were not assigned to any specific genes and were categorized as “Unassigned_NoFeatures: alignments that do not overlap any feature” by the featureCounts analysis (Additional file 4: Table S4). Possible reasons for those reads not being assigned to any genes in the references include but may not be limited to, i) incomplete annotation of the reference genomes, ii) novel transcripts, iii) small amount of DNA contamination in the RNA samples, and iv) other sequencing artifacts. Characterization of the hypothetical proteins and exploration for potential novel transcripts may be necessary to completely understand the mechanism of *S. enterica* interaction with bovine epithelial cells.

### Genes located on potential SPIs and virulence factors

**Fig. 2:**
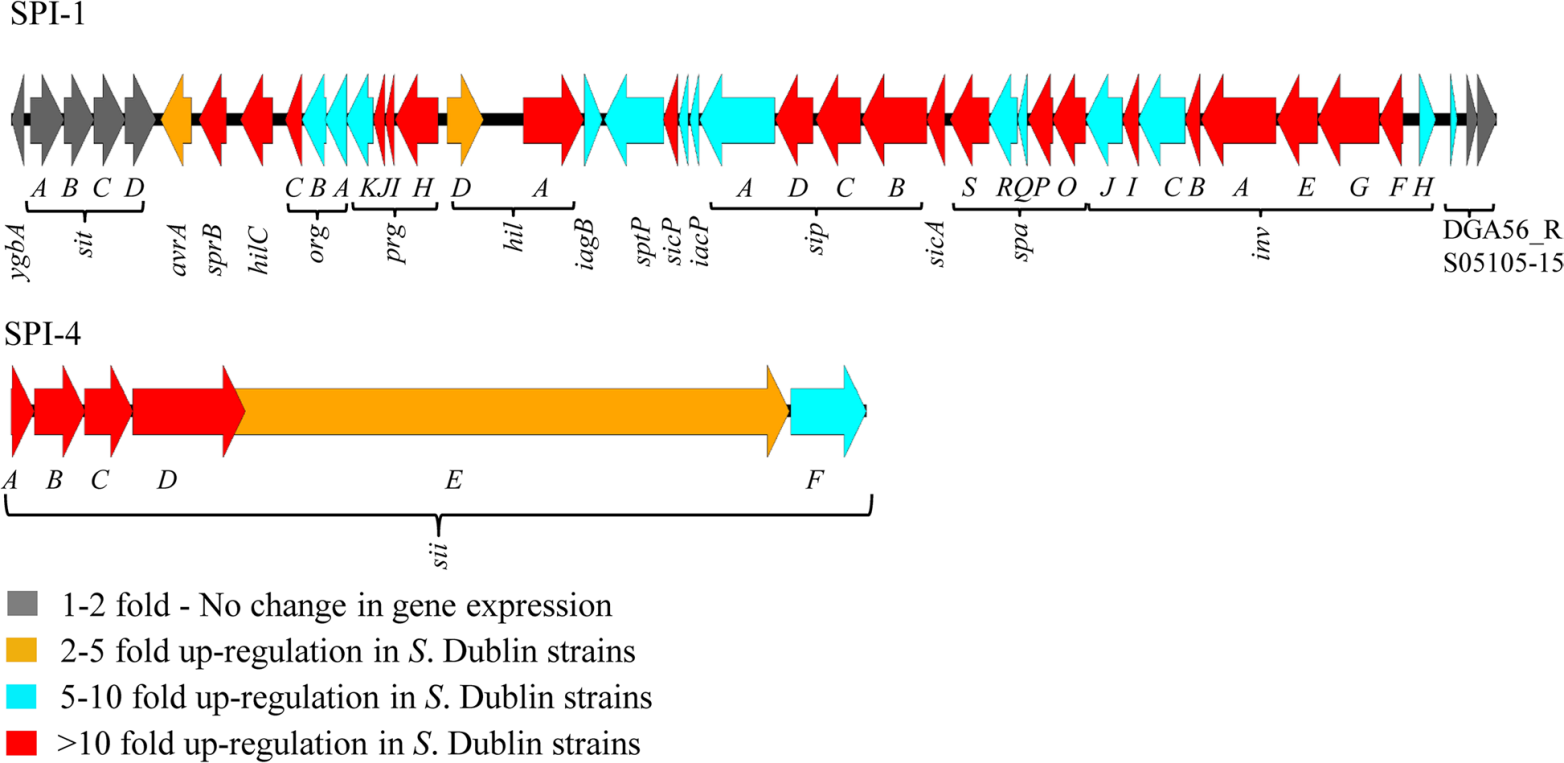
The relative expression of genes in two pathogenicity islands, SPI-1 and SPI-4, of *S*. Dublin strains compared with *S*. Cerro strains during interaction with bovine epithelial cells. SPIs are scaled against each other. Each horizontal arrow represents a gene to scale within an SPI. The color of each arrow represents relative expression level in the *S*. Dublin strains compared with the *S*. Cerro strains while they were associated with bovine epithelial cells

*S. enterica* genomes contain SPIs that are generally large gene cassettes and are known contributors of infection outcomes in various hosts. The numbers of SPIs harbored by *S. enterica* strains may be highly variable and serovar-dependent. Hsu et al. [32] identified 15 SPIs among 69 *S*. Dublin strains isolated from sick cattle, retail beef, and humans in the United States and Zhao et al. [44] identified 10 SPIs in two *S*. Cerro strains isolated from diseased animals. When *S*. Dublin and *S*. Cerro strains were interacting with bovine epithelial cells, *S. enterica* genes in the SPI-regions that were upregulated in the *S*. Dublin strains compared with the *S*. Cerro strains included: 37 SPI-1 genes encoding mostly T3SS apparatus and effectors; all of the six SPI-4 genes encoding type I secretion apparatus (*siiABCDEF*); T3SS effectors and chaperone (*sopB*, *pipB*, and *sigE*) located in SPI-5; type VI secretion system (T6SS) associated protein coding genes (*sciJKNOR*) located in SPI-6; and T3SS effector *sopF* in SPI-11 (Fig. 2).

SPI-1 encoded T3SS is one of the most important virulence factors of *S. enterica*. SPI-1 encoded gene products have important roles in the invasion of host epithelial cells by *S. enterica* cells, as well as in the regulation/modulation of host immune response (e.g., induce recruitment of neutrophils), and biofilm formation [45]. Shah et al. [28] reported that 38 genes of SPI-1 were upregulated in *S*. Enteritidis strains from “high-pathogenicity group” compared with their “low-pathogenicity group” counterparts. Clark et al. [46] reported that differences in *S*. Typhimurium invasiveness to Madin-Darby Canine Kidney cells were associated with heterogenous expression of genes located on SPI-1. In addition to SPI-1 genes, the genes encoding other SPI-1 T3SS-translocated effectors (e.g., SopB encoded by SPI-5, SopF encoded by SPI-11, SopA and SopD encoded elsewhere in the *S*. Dublin genome) were up-regulated (by 4 to 58-fold, median = 19-fold) in the *S*. Dublin strains compared with the *S*. Cerro strains, concurring with previous work indicating that genes encoding effectors that are translocated via SPI-1 T3SS have an SPI-1-like expression pattern [41].

Significant upregulation of SPI-1 genes and SPI-1 T3SS-effectors in the *S*. Dublin strains may have promoted the higher level of invasion compared with the *S*. Cerro strains in bovine epithelial cells that was observed with the same strains in a previous study [13]. The regulation of SPI-1 gene expression in *S. enterica* involves a complex network of transcription regulators [47] but the reason for the observed heterogeneity in SPI-1 gene expression in strains from different serovars is not known. A critical part of the SPI-1 regulatory network is the feed-forward regulatory loop of HilC-RtsA-HilD, where *hilC* and *hilD* genes are located within the SPI-1 and *rtsA* is located approximately 3 Mbp downstream of the SPI-1 in the *S*. Dublin and *S*. Cerro genomes. We observed more than a 30-fold increased expression of *hilC* and *rtsA* genes and more than a 3-fold increased expression of *hilD* gene in the *S*. Dublin strains compared with the *S*. Cerro strains. These higher levels of expression may have driven the increased expression of SPI-1 genes in the *S*. Dublin strains. However, we did not observe differential expression of *hilE*, an important negative regulator of SPI-1 which down-regulates the expression of the SPI-1 genes by inactivating HilD [48]. In addition, we did not observe differential expression of other SPI-1 regulators (e.g., global regulator of carbohydrate metabolism, Mlc; two-component regulatory system, BarA/SirA; global regulatory RNA binding protein, CsrA; protease, Lon; Histone-like nucleoid-structuring protein, H-NS) that directly or indirectly influence SPI-1 expression [47]. Jiang et al. [49] reported that a regulator encoded within SPI-14, LoiA (low oxygen induced factor A), activates transcription of SPI-1 positive regulator, *hilD*, and that deletion of either the entire SPI-14 region or the single *loiA* gene dramatically attenuated *S*. Typhimurium virulence. Using RNA-Seq technology, Li et al. [50] observed that SPI-1 gene expression was down-regulated in a *S*. Typhimurium *loiA* mutant strain. In accordance with previous literature, in this study, SPI-14 was uniquely identified in the *S*. Dublin strains but not in the *S*. Cerro strains [32, 44]. The presence of SPI-14 in the *S*. Dublin strains may have had an impact on the increased expression of SPI-1 genes in the *S*. Dublin strains compared with the *S*. Cerro strains.

All of the six SPI-4 genes (*siiABCDEF*) encoding a type I secretion apparatus and a non-fimbrial adhesin, SiiE, were upregulated by 4 to 56-fold (median: 22-fold) in the *S*. Dublin strains compared with the *S*. Cerro strains. SiiE is expressed on the surface of *S. enterica* cells and is responsible for the adhesion of *Salmonella* to epithelial cells [51]. SiiE is important for *S.* Typhimurium invasion of bovine enterocytes and colonization in cattle, and its expression is directly influenced by the SPI-1 positive regulator, HilA [52]. Cooperative action of SPI-1 and SPI-4 promotes a strong attachment of *Salmonella* to the epithelial barrier and facilitate efficient SPI-1 mediated translocation [53]. In this study, the upregulation of HilA in the *S*. Dublin strains compared with the *S*. Cerro strains may have promoted the expression of SPI-4 by countering H-NS-mediated silencing [54] and that may have played a role in the observed higher association of the *S*. Dublin vs the *S*. Cerro strains with bovine epithelial cells.

### Fimbriae-associated genes

Fimbriae are primary organelles that play an important role in *S. enterica* virulence by allowing interaction with, and adherence to the host intestinal epithelium [55]. There are multiple fimbrial clusters in *S. enterica* and the *fim* cluster that encodes a type 1 fimbriae (T1F) is highly conserved among *S. enterica* strains [56]. The *fim* cluster in *S. enterica* comprises 10 genes, six (*fimAICDHF*) of which encode proteins that are involved in the biogenesis of T1F. When grown in LB broth, *S*. Typhimurium expressed only FimA (among other putative major fimbrial subunits: AgfA, FimA, PefA, LpfA, BcfA, StbA, StcA, StdA, StfA, SthA, and StiA), whereas the same strain expressed the majority of those fimbrial subunits when injected into bovine ligated ileal loops [57]. In this study, when co-cultured with bovine epithelial cells, all six genes encoding T1F biogenesis proteins were upregulated in the *S*. Cerro strains (by 5 to 23-fold) compared with the *S*. Dublin strains.

FimW, a major regulatory protein located within the *fim* cluster represses T1F expression [58]. In this study, *fimW* was upregulated in the *S*. Dublin strains by more than 2-fold explaining the lower level of T1F expression observed in the *S*. Dublin strains compared with the *S*. Cerro strains. This difference in T1F expression indicates that the *S*. Dublin strains may rely on other adhesins for attachment to bovine epithelial cells. Indeed, unlike *S*. Cerro strains, *S*. Dublin strains harbored a long polar fimbriae cluster, *lpf*, which has been shown to mediate adhesion of *S*. Typhimurium to murine Peyer’s patches [59]. The role of Lpf on the interaction of *S*. Dublin with bovine epithelial cells is not known.

Significant upregulation (> 4-fold) of CsgDEFG from the curli subunit gene, *csg*, operon was observed in the *S*. Dublin strains (TPM range: 26 to 142) compared with the *S*. Cerro strains (TPM range: 5.7 to 30.1). Shah et al. [27] described upregulation of CsgC in highly pathogenic *S*. Enteritidis strains compared with less pathogenic strains. Csg are fimbrial adhesins that help *S. enterica* to auto-aggregate, adhere to surfaces, and form biofilms [60]. However, the specific role of Csg in *S*. Dublin or *S*. Cerro pathogenicity towards the bovine host is also not known. Further studies are necessary to understand the roles of a diverse profile of fimbriae in *S. enterica* serovars while they are interacting with bovine host cells.

### Metabolic pathways (Fatty acid *β*-oxidation)

Genes that were differentially expressed between *S*. Dublin and *S*. Cerro strains (P_*adj*_ < 0.05) or unique to one of the serovars were mapped to the metabolic pathways listed in the KEGG database. When distribution of these genes in different pathways were manually inspected, it appeared that proteins encoded by DEGs/serovar-specific unique genes occupied a small percentage of all possible proteins that facilitate most of the pathways except for the fatty acid *β*-oxidation pathway.

*S. enterica* genomes contain the complete set of *β*-oxidation genes for fatty acid catabolism (*fad*) and *S*. *enterica* can use fatty acids as unique carbon sources [61, 62]. Saturated fatty acids that are routed into the *β*-oxidation pathway are converted to acetyl-CoA that can be routed into the tricarboxylic acid (TCA) cycle for complete catabolism and release of energy. Reens et al. [63] observed that *S. enterica* requires lipid metabolism genes to replicate in mouse macrophages; lipid import, and *β*-oxidation genes were required for *S. enterica* colonization in mouse tissue. It has also been reported that *S. enterica* may take up fatty acids from the *Salmonella*-containing vacuole (SCV) and degrade them via the *β*-oxidation pathway [64]. The fatty acid metabolism/requirement of *S. enterica* during bovine colonization/infection is currently unknown. When we explored the genes associated with the core cycle of the fatty acid *β*-oxidation I pathway [65] of *S. enterica* strains associated with bovine epithelial cells, most of the *β*-oxidation pathway genes were upregulated/uniquely present in the *S*. Dublin strains compared with the *S*. Cerro strains. For example, an accessory acyl-CoA synthase (gene: *fdrA*, gen id: DZA56_RS18785, average TPM in *S*. Dublin strains: 28.91) and an accessory acyl-CoA dehydrogenase (gene id: DZA56_RS16850, average TPM in *S*. Dublin strains: 20.65) were identified in the *S*. Dublin genomes but not in the *S*. Cerro genomes. In addition, several genes from the *β*-oxidation pathway (e.g., *fadB*, *fadI*, and *fadJ*) were upregulated by 1.76 to 2.52-fold (P_*adj*_ < 0.05) in the *S*. Dublin strains compared with the *S*. Cerro strains. These findings indicate that *S*. Dublin strains may have an altered proficiency in metabolizing fatty acids compared with the *S*. Cerro strains. We hypothesize that an altered lipid metabolism may have played a role in the observed differential associations of *S*. Dublin and *S*. Cerro strains with bovine epithelial cells. Further studies are necessary to validate potentially altered lipid metabolism in *S*. enterica serovars to determine its role on interaction between *S*. *enterica* and bovine host cells.

## Conclusion

Higher levels of association and invasiveness of *S*. Dublin strains in bovine epithelial cells compared with *S*. Cerro strains appears to be due to a complex set of factors: i) significant upregulation of SPI-1 genes and T3SS effectors in the *S*. Dublin strains (the presence of SPI-14 among *S*. Dublin strains is suggested to play a role in SPI-1 gene regulation), ii) upregulation of SPI-4 genes encoding Type I secretion apparatus potentially due to the upregulation of SPI-1 genes, iii) altered profile and expression of fimbriae associated genes, and iv) differential proficiency to metabolizing fatty acids due to accessory genes and/or DEGs. However, all of the above-mentioned factors need to be experimentally confirmed since gene expression does not always lead to protein expression. Results also indicate that presence/absence of a gene in *S. enterica* serovars may not completely explain a phenotype due to possible differential expression of that gene in different serovars. Therefore, presence/absence of a suite of genes may need to be considered rather than a single gene to explain an observed phenotype. This study identifies genes of *S. enterica* that may be responsible for symptomatic or asymptomatic infection/colonization of two bovine-adapted serovars in cattle. Additionally, the observed differences in gene expression between strains of *S*. Dublin and *S*. Cerro may identify specific targets that can be leveraged to reduce occurrences of the common food safety pathogen, *S*. *enterica*, in dairy animals.

## Supplementary Information

**Additional file 1. Table S1.** Preparation of a comparison matrix for differential expression analysis.

**Additional file 2. Table S2.** List of differentially expressed genes (at least 2-fold differences) in the transcript abundances between strains from the two serovars with a false discovery rate ≤ 5% using DESeq2 and edgeR. TPM, Transcripts Per Kilobase Million.

**Additional file 3. Table S3.** Predicted signal peptides among the translated proteins of uncategorized DEGs.

**Additional file 4. Table S4.** List of sequence identifiers from the “Unassigned NoFeatures” category.

## Data Availability

The datasets supporting the conclusions of this article are available in the Gene Expression Omnibus (GEO; https://www.ncbi.nlm.nih.gov/geo/) under accession number GSE182165.
