# Supplementary material for: Differences between the global transcriptomes of Salmonella enterica serovars Dublin and Cerro infecting bovine epithelial cells
Source: BMC Genomics. 2022 Jul 8;23:498. doi: 10.1186/s12864-022-08725-z (PMC9270791; doi:10.1186/s12864-022-08725-z)
Supplement: Supplementary file 1 — Additional file 1. Table S1. Preparation of a comparison matrix for differential expression analysis. [file 12864_2022_8725_MOESM1_ESM.docx]

**Additional file 1: Table S1: Preparation of a comparison matrix for differential expression analysis**

| 1. Coding sequences of the two reference genomes (*S. enterica* serovar Dublin strain USMARC-69838 chromosome and plasmid pSDU1-USMARC-69838 - GenBank accession numbers: NZ_CP032449.1, NZ_CP032450.1; *S. enterica* serovar Cerro strain CFSAN001588 chromosome and plasmids pCFSAN001588_001 and pCFSAN001588_002 - GenBank accession numbers: NZ_CP012833.1, NZ_CP012834.1, NZ_CP012835.1) were clustered with get_homologues_est (Contreras-Moreira and Vinuesa, 2013) using minimum sequence similarity (-s) of 80% and coverage (-c) of 80%. Each of the gene clusters from the get_homologues_est output were inspected manually; two genes from a cluster were considered to be gene homologues if, i) length of one gene was ≥ 95% of the length of the other gene, and ii) both were annotated as same gene name by the NCBI Prokaryotic Genome Annotation Pipeline (PGAP). |
| --- |
| 1. Genes that were not clustered using the above-mentioned method, translated protein sequences of those genes were clustered using BlastRules (BlastRuleException: amino acid identity ≥ 94%, model protein coverage ≥ 90%, target protein coverage ≥ 90%). BlastRules are a type of evidence for functional classification of proteins and is used by the PGAP (<https://www.ncbi.nlm.nih.gov/genome/annotation_prok/evidence/>). |
| 1. Lastly, a few numbers of gene homologues from the two reference genomes were determined by manual curation. Gene pairs were considered functionally similar if they were annotated as same gene name by the PGAP, and their flanking genes (in respective genomes) were identified as homologues using both of the two methods mentioned above. For example, there were only 77% similarity in the nucleotide sequences of the *fliD* genes from the two reference genomes identified using the MEGABLAST algorithm via the National Center for Biotechnology Information (NCBI) web server ( <http://www.ncbi.nlm.nih.gov/>). However, genes that flanked the *fliD* gene in the two reference genomes were found to be homologous using both of the above-mentioned methods. Therefore, *filD* genes from the *S*. Dublin and *S*. Cerro reference genomes were considered homologues. |
